# The predictive and diagnostic accuracy of long pentraxin-3 in COVID-19 pneumonia

**DOI:** 10.3906/sag-2011-32

**Published:** 2021-04-30

**Authors:** Ahmed Bilal GENÇ, Selçuk YAYLACI, Hamad DHEİR, Ahmed Cihad GENÇ, Kubilay İŞSEVER1, Deniz ÇEKİÇ1, Havva KOCAYİĞİT, Erdem ÇOKLUK, Alper KARACAN, Mehmet Ramazan ŞEKEROĞLU, Hande TOPTAN ÇAKAR, Ertuğrul GÜÇLÜ5

**Affiliations:** 1 Department of Internal Medicine, Faculty of Medicine, Sakarya University, Sakarya Turkey; 2 Department of Intensive Care, Faculty of Medicine, Sakarya University, Sakarya Turkey; 3 Department of Biochemistry, Faculty of Medicine, Sakarya University, Sakarya Turkey; 4 Department of Radiology, Faculty of Medicine, Sakarya University, Sakarya Turkey; 5 Department of Microbiology, Faculty of Medicine, Sakarya University, Sakarya Turkey

**Keywords:** COVID-19, SARS-CoV-2, pentraxin, disease progression, mortality

## Abstract

**Background/aim:**

The purpose of this study is to evaluate serum pentraxin-3 (PTX-3) levels in Sars-CoV-2 virus infection (COVID-19) patients and to investigate whether PTX-3 predicts the disease prognosis.

**Materials and methods:**

This study was conducted on 88 confirmed COVID-19 patients who were hospitalized due to symptomatic pneumonia between April 15 and August 15, 2020. The patients were divided into two groups as survived patients and non-survived patients. Both groups were compared according to demographic features, comorbid conditions and measurement of the PTX-3 and other laboratory parameters of the patients.

**Results:**

Of 88 patients with COVID-19, 59 (67%) were discharged with complete cure and 29 (33%) resulted in death. 46 (52.3%) of the patients were men. PTX-3 median value (IQR) was 3.66 ng/mL (0.9–27.9) in all patients, 3.3 ng/mL (0.9–27.9) in survivors and 3.91 ng/mL (1.9–23.2) in nonsurvivors which was significantly higher (P = 0.045). As a receiver operating characteristic curve analysis the cut-off value of PTX-3 for predicting mortality in patients was 3.73 with 65% sensitivity and 65% specificity (AUC: 0.646, 95% CI: 0.525–0.767, P = 0.045). Also, we found significant cut-off values with respect to D-dimer, D-dimer/PTX-3, high-sensitivity troponin, high-sensitivity troponin/PTX-3, lymphocyte, PTX-3/lymphocyte, procalcitonin, procalcitonin/PTX-3, CRP, and CRP/PTX-3 (P < 0.05).

**Conclusion:**

In this study, as far as we know, for the first time, we have shown PTX-3 as the new mortality biomarker for COVID-19 disease.

## 1. Introduction

The new coronavirus outbreak is the worst disaster for humanity in the twenty-first century. According to the data of the World Health Organization (WHO), above forty seven million cases were detected and more than 1 million deaths from COVID-19 disease were reported. Fortunately, most patients with COVID-19 can be cured and far fewer infected people can result in death. 

In a study included 44,672 confirmed cases. Among confirmed cases, most were aged 30–79 years (86.6%), and considered mild (80.9%). A total of 1023 deaths occurred among confirmed cases for an overall case fatality rate of 2.3% [1].****

The presence of COVID-19 may be asymptomatic or present with mild/moderate or severe symptoms. Based on clinical manifestations, blood tests, and computerized tomography, this disease was diagnosed as virus-induced pneumonia by clinicians. Now the majority of cases can be diagnosed by reverse transcription-polymerase chain reaction (RT-PCR) nasopharyngeal (NP) swabs [2]. Common symptoms include cough, fever, and shortness of breath. Other reported symptoms are weakness, malaise, respiratory distress, muscle pain, sore throat, loss of taste and/or smell [3]. Many biomarkers have been shown in terms of evaluating these complaints and investigating whether they provide clear information about the disease prognosis [4]. Perhaps the most important of these indicators is C-reactive protein (CRP) and it has been proven that it can determine the prognosis of COVID-19 disease [4,5]. CRP –in addition with serum amyloid A– represents the short arm of the N-terminal area of pentraxins and produced in the liver under the stimulatory influence of interleukin-6 (IL-6). Long pentraxins are neuronal pentraxin 1 (NP1), neuronal pentraxin 1 (NP2), pentraxin-3 (PTX-3), pentraxin-4 (PTX-4) and neuronal pentraxin receptor [6]. 

PTX-3 was the first long pentraxin discovered 30 years ago. It has induced by interleukin-1 beta (IL-1beta) and TNF-alpha in some cells such as fibroblasts, epithelial cells and endothelial cells [7]. Also, PTX3 produced in human antigen-presenting cells, such as monocytes, macrophages, and dendritic cells, can be induced by viral and nonviral infectious ajanets [7–10]. In animal studies, the administration of recombinant PTX-3 against various non-COVID-19 infectious agents has been shown as a full protective or adjunctive therapeutic feature from acute lung injury [10]. According to these results, serum PTX-3 elevation may also indicate acute pulmonary damage in COVID-19 disease. The purpose of this study is to evaluate serum PTX-3 levels in COVID-19 patients and to investigate whether PTX-3 determines the disease prognosis.

## 2. Materials and methods

This study was conducted on 88 confirmed COVID-19 patients who were hospitalized due to symptomatic pneumonia between April 15 and August 15, 2020. The study was conducted in accordance with the Declaration of Helsinki. The study population was determined as patients hospitalized in the training and research hospital within the specified period. Patients whose serum could be separated for PTX-3 at the time of hospitalization were included in the study. Also, patients with symptomatic pneumonia, had indication for hospitalization and had confirmation of COVID-19 by reverse transcription-polymerase chain reaction (RT-PCR) nasopharyngeal (NP) swabs were consecutively enrolled. The patients who did not have radiologic signs of pneumonia, NP RT-PCR negative, have malignancy, have confirmed bacterial infection at admission were excluded. The patients were divided into two groups as survived patients (group 1) and nonsurvived patients (group 2). Both groups were compared according to demographic features, comorbid conditions and measurement of the laboratory parameters of the patients. Before receiving any antimicrobial or antiinflamatuar drug, the serum PTX-3’s were obtained from all patients at the first admission and studied on the same day.****

Pentraxin-3 levels: Human pentraxin-3 levels were analyzed by sandwich model double antibody enzyme-linked immunoabsorbent method with a commercial human ELISA kit from Bioassay Technology Laboratory (BT Laboratory Co., Ltd., Shanghai, China). Results are expressed as ng/ml. The within-run and between-run CV% of the analytes were given as <10%, and the measurement range was specified as 0.1–30 ng/mL.

### 2.1. Statistical analysis 

Descriptive analyses were performed to provide information on general characteristics of the study population. Kolmogorov–Smirnov/Shapiro–Wilk’s tests were used to determine whether or not they are normally distributed. Descriptive analyses were presented using medians, minimum-maximum values and interquartile range (IR) for the nonnormally distributed variables. The Mann–Whitney U test was used for nonparametric tests to compare these parameters. Chi-square test used to compare the categorical variables between two groups. The categorical variables were presented as the frequency (% percentage). The performance of PTX-3, high-sensitivity troponin, procalcitonin, LEU, C-reactive protein and pentraxin ratio of these was assessed using receiver operating characteristic (ROC) curve analysis and by calculating the area under the curve (AUC) of the ROC curves. A P-value <0.05 was considered significant. Analyses were performed using SPSS statistical software (IBM SPSS Statistics, version 22.0, IBM Corporation, Armonk, NY, USA).

## 3. Results

Of 88 patients with COVID-19, 59 (67%) were discharged with complete cure and 29 (33%) resulted in death. 42 of the patients (47.7%) were women and 46 (52.3%) were men. Of those who died, 12 (41.4%) were women, 17 (58.6%) were men, and there was no significant difference in mortality between men and women (P = 0.403). In terms of age compared according to mortality, the median (IQR) age of the survivors was 61 (23–92), while the median age of the deceased group was 72 (50–95) (P < 0.001). In terms of comorbidities there was no significant difference between survivor and nonsurvivor patients **(** Table 1). The most common symptoms in all patients were cough (48.90%), shortness of breath (45.5%), and fever (42.0%). Complaints such as myalgia, anosmia, acute gastroenteritis, and weakness were less frequent (18.20%). Considering the symptoms at the time of admission to the hospital, fever of >37.5 **°** C was significantly more prominent in the patients who survived (respectively; 52.50 vs. 20.70%; P = 0.006), while shortness of breath was more prominent in patients who did not experience (32.20% vs. 72.40%; P = 0.000). Significant differences were found between the two groups in hematological and biochemical measurements, which are among the mortality indicators of COVID-19 disease (Table 2). PTX-3 median value (IQR) was 3.66 ng/mL (0.9–27.9) in all patients, 3.3 ng/mL (0.9–27.9) in survivors and 3.91 ng/mL (1.9–23.2) in nonsurvivors which was significantly higher (P = 0.045) (Table 2).

**Table 1:** Demographic characteristics of patients with COVID-19.

Items	All patientsno = 88	Survivorsno = 59	Nonsurvivorsno =29	P
Age (years, min-max)	67 (23–95)	61 (23–92)	72 (50–95)	0.000
Sex (male/female), no, %	46/42 (47.70/(52.30)	29/30 (49.20/50.80)	17/12 (58.60/41.4)	0.403
Comorbidities, no, % Diabetes mellitus Hypertension Chronic heart disease Chronic obstructive pulmonary disease	28 (31.80) 45 (51.10) 18 (20.70) 6 (6.80)	22 (37.30) 30 (50.80) 10 (17.20) 4 (6.80)	6 (20.70) 15 (51.70) 8 (27.60) 2 (6.90)	0.116 0.938 0.261 0.989
Symptoms at admission no,% Fever Cough Shortness of breath Others	37 (42.00) 43 (48.90) 40 (45.50) 31 (35.20)	31 (52.50) 31 (52.50) 19 (32.20) 24 (40.70)	6 (20.70) 12 (41.40) 21 (72.40) 7 (24.10)	0.006 0.325 0.000 0.127
Supporting/antiviral treatments, no, % Hydroxyqlorochine Favipiravir Antibacterial antibiotics Others	85 (96.60) 55 (62.50) 61 (69.30) 16 (18.20)	56 (94.90) 33 (55.90) 43 (72.90) 7 (11.90)	29 (100.00) 22 (75.90) 18 (62.10) 18 (62.10)	0.548 0.069 0.301 0.028
Hospitalization at Ward Hospitalization at ICU	46 (52.30%) 42 (47.70%)	46 (78.00%) 13 (22.00%)	0 (0%) 29 (100%)	0.000

**Table 2:** Comparison of hematological and biochemical parameters between patients with COVID-19.

Characteristics	All patientsno = 88	Survivorsno = 59	Nonsurvivorsno = 29	P
WBC, K/uL	6.4 (2.4–31.4)	6 (2.4–31.4)	9.1 (2.9–26)	0.003
Neutrophil, K/uL	4.8 (1.7–29)	3.9 (1.7–29)	6.7 (1.7–22.1)	0.004
Lymphocyte, K/uL	1.04 (0.3–6.1)	1.21 (0.4–6.1)	0.774 (0.3–3.2)	0.001
Hemoglobin, g/dL	12.7 (7–16.5)	12.7 (9.6–16)	12.6 (7–16.5)	0.650
Platelet count, K/uL	178.5 (74.1–717)	176 (85.4–589)	179 (74.1–717)	0.814
Prothrombin time, s	13 (10–26.9)	12 (10–26.9)	3.3 (10.4–16.8)	0.007
AST, U/L	33 (15–291)	32 (16–291)	44 (15–159)	0.063
ALT, U/L	23 (7–292)	23 (7–292)	22 (7–135)	0.378
Serum albumin, g/L	3.3 (1.9–4.2)	3.4 (2–4.2)	3 (1.9–3.6)	0.000
hsTn I, ng/L	8 (0.5–1124)	5.5 (0.5–273)	1210 (135–38800)	0.000
Total cholesterol, mg/dL	153 (73–248)	159 (96–248)	145 (73–240)	0.021
LDL-cholesterol, mg/dL	100 (44–188)	102 (53–188)	100 (44–188)	0.009
Ferritin, ug/L	425 (35–6321)	281 (35–6321)	653 (136–4625)	0.001
D-dimer, ugFEU/L	671.5 (89–38800)	516 (89–34200)	1210 (135–38800)	0.000
LDH, U/L	335 (126–994)	291 (143–718)	429.5 (126–994)	0.000
CRP, mg/L	75.15 (1–386)	44.5 (1–386)	141 (4.9–356)	0.000
Procalcitonin, ng/mL	0.157 (0–100)	0.082 (0–100)	0.408 (0.1–100)	0.000
Lactat, mmol/L	1.7 (0.7–4.3)	1.6 (0.7–3.7)	1.9 (1–4.3)	0.016
Fibrinojen, mg/dL	373 (154–746)	337 (166–746)	434 (154–542)	0.016
Serum PTX-3 at admission (ng/mL)	3.66 (0.9–27.9)	3.3 (0.9–27.9)	3.91 (1.9–23.2)	0.045

ROC analysis was performed to determine whether the value of pentraxin had a significant cut-off value in predicting mortality in patients with a diagnosis of COVID-19. The optimal cut-off value of pentraxin for predicting mortality in patients was 3.73 with 65% sensitivity and 65% specificity (AUC: 0.646, 95% CI: 0.525–0.767, P = 0.045). Likewise, significant cut-off values were determined for D-dimer, D-dimer/PTX-3, high-sensitivity troponin, high-sensitivity troponin/PTX-3, 1/lymphocyte, PTX-3/lymphocyte, procalcitonin, procalcitonin/PTX-3, CRP, and CRP/PTX-3 (P < 0.05) (Table 3) (Figure) . 

**Table 3:** Diagnostic performance of single and combined biochemical indicators on differentiating survivor and nonsurvivor patients with COVID-19.

Variables	Cut-off value	Sensitivity	Specificity	AUC	95% CI	P
PTX-3	3.73	65%	65%	0.646	(0.525–0.767)	0.045
D-dimer	833	73%	68%	0.752	(0.643–0.861)	0.000
D-dimer/PTX-3	282	66%	66%	0.676	(0.545–0.807)	0.012
Troponin	11	80%	80%	0.830	(0.734–0.925)	0.000
Troponin/PTX-3	2.45	77%	74%	0.769	(0.655–0.883)	0.000
1/Lymphocyte	1	69%	64%	0.699	(0.565–0.833)	0.004
PTX-3/Lymphocyte	3.8	69%	68%	0.726	(0.608–0.843)	0.001
Procalcitonin	0.205	80%	78%	0.821	(0.726–0.917)	0.000
Procalcitonin/PTX-3	0.05	81%	72%	0.770	(0.660–0.880)	0.000
C-reactive protein	110.5	69%	73%	0.738	(0.63–0.845)	0.000
C-reactive protein/PTX-3	27.7	69%	70%	0.679	(0.561–0.796)	0.007

**Figure:**
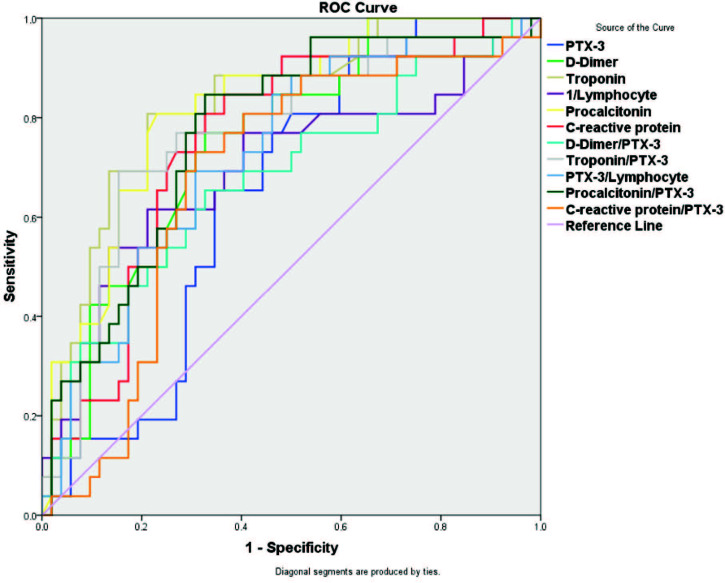
ROC curves of pentraxin-3 and combined blood biomarkers on differentiating survive patients from nonsurvives with COVID-19.

## 4. Discussion

In this study, as far as we know, for the first time, we have shown pentraxin-3 as the new mortality biomarker for COVID-19 disease. Some hematological and inflammatory indicators have been identified as factors associated with the death of patients with COVID-19 pneumonia caused by the novel coronavirus SARS-CoV-2 [11]. Measurement of acute phase proteins, such as C-reactive protein, is routinely used for diagnosis and determining the prognosis of infectious disease [12]. Previous studies, however, demonstrated that C-reactive protein is unlikely to be useful clinically in non-COVID viral infections or disease severity [13]. In a metaanalysis for acute phase reactants related to COVID-19 revealed that elevated serum CRP was associated with an increased some poor outcome [RR 1.84 (1.45, 2.33), P < 0.001; I2: 96%, P < 0.001] like severity of disease or need for intensive care unit, but not mortality [14]. Differences have been shown between COVID-19 studies regarding the significance of sensitivity of CRP values in determining disease prognosis. This metaanalysis determined a single cut-off point of ≥10 mg/L for CRP resulted in a sensitivity of 51% (18%–84%) and a specificity of 88% (70%–95%). [14].  However, in another metaanalysis, in mortal COVID-19 patients, compared to those who survived, there was a significant difference with respect to CRP and other hematologic and inflammatory biomarkers (P < 0.05) [11]. Likewise, in line with the literature information, we identified a correlation between acute phase reactants and inflammatory biomarkers including leukocytosis, lymphopenia, CRP, cardiac troponin, D-dimer, fibrinogen, lactate dehydrogenase, ferritin, and serum albumin and mortality. In addition, we found a significant relationship between serum PTX-3 levels, which is an indicator of inflammation, and death due to COVID-19 pneumonia. Also, by using ROC analysis we found the closest cut-off value of CRP as ≥110.5 mg/L resulted in sensitivity of 69% and specificity of 73%. When various cut-off values for CRP shown in many studies [15–17] are analyzed on our data; for CRP ≥10 mg/ L as cut-off value, sensitivity and specificity rates were 93% and 25%, respectively, while for CRP ≥40 mg/ L cut-off value, these rates were 93% and 45%, respectively. 

In non-COVID-19 prospective controlled study conducted on 142 septic, septic shock and healthy individuals revealed that both the initial and follow-up PTX3 levels were consistently significantly higher in patients who died than in those who recovered (initial P = 0.004; follow-up P < 0.001). The optimal cut-off value of serum PTX3 to discriminate sepsis from healthy individuals was 15.10 ng/mL (sensitivity, 92.6%; specificity, 97.4%; P < 0.001) and distinguish septic shock was 58.28 ng/mL (93.2% sensitivity, 60.7% specificity, P < 0.001). Consequently, the increase of serum PTX-3 levels in critically ill patients was correlated with the severity of the diseases from systemic inflammatory response syndrome to septic shock and sepsis [18].

As general information, while PTX-3 levels increase after 6–8 h of inflammatory process and may result in the release of certain cytokines that may cause a cytokine storm, it is necessary to wait more than one and half days for an increase in CRP levels to respond to inflammation. The levels of PTX-3 in the sera are very low in normal individuals (<2 ng/mL), which are rapidly and extremely increased in patients with various infectious and inflammatory status [19]. Considering that acute respiratory distress syndrome due to COVID-19 pneumonia develops very aggressively and rapidly, it can be said that measuring serum long PTX-3 levels has been proposed to be a marker for early diagnosis in order to predict the progression of the disease.

Our study has some limitations including investigating the PTX-3 levels only on the day of admission. In fact, it can be said that if we had taken serum PTX-3 samples during hospitalization or when patients progressed, we might have detected higher PTX-3 levels. Also, it does not include a healthy control group.

In conclusion, our study included a cohort of COVID-19 patients with a mortality rate of 32.9%. Our study mortality rate is very high. Because of we are 3rd step center, patients with relatively severe pneumonia were hospitalized. In addition to the classical predictors of mortality reported in the literature, we showed that the length PTX-3 –as a specific marker of vascular inflammation– could be a new marker of mortality associated with COVID-19. There is no general agreement on a cut-off point of CRP to determine the severity of COVID-19. Due to the worldwide outbreak of the COVID-19 pandemic, and in an environment where there is no definitive vaccine or treatment approach yet, we think that biomarkers such as PTX-3 may determine the prognosis of the disease.

## Conflict of interest

The authors declare that they have no conflict of interest.

## Informed consent

The ethics committee approval from Sakarya University Faculty of Medicine was provided for this study (No: 71522473/050.01.04/460).

No written informed consent was necessary for this type of study. The data used to support the findings of this study are available from the corresponding author upon request.
